# Effects of Fly Maggot Protein Replacement of Fish Meal on Growth Performance, Immune Level, Antioxidant Level, and Fecal Flora of Blue Foxes at Weaning Stage

**DOI:** 10.3390/ani12121480

**Published:** 2022-06-07

**Authors:** Yuan Xu, Hang Su, Ting Li, Jing Lv, Jiayu Liu, Xiujuan Bai

**Affiliations:** 1 College of Animal Sciences and Technology, Northeast Agricultural University, Harbin 150030, China; yuanxu@neau.edu.cn (Y.X.); suhang609@126.com (H.S.); s200501025@neau.edu.cn (T.L.); lvjing@neau.edu.cn (J.L.); 2 Branch of Animal Husbandry and Veterinary of Heilongjiang Academy of Agricultural Sciences, Qiqihar 161005, China

**Keywords:** *Alopex lagopus*, fishmeal replacement, insect protein, average daily gain, health, 16S rRNA high-throughput amplicon target sequencing

## Abstract

**Simple Summary:**

Given the shortage of fish meal, other animal protein replacements are actively being researched. One such alternative is fly maggot protein—a highly nutritious insect protein containing chitin, lysozyme, and other biologically active substances. The purpose of this study was to examine how the replacement of fish meal with fly maggot protein affects growth, immune indexes, antioxidant levels, and fecal microflora in blue foxes (*Alopex lagopus*) during weaning. The results showed that fly maggot protein replacement had no adverse effects on the growth, immune indexes, and antioxidant levels of blue foxes at weaning. Further, it could modulate the fecal microflora. This indicates that fly maggot protein can serve as a novel animal protein alternative to fish meal for feeding blue foxes.

**Abstract:**

Dietary protein is a key nutritional parameter and warrants special attention in animal husbandry. This study aimed to evaluate the effect of replacing fish meal (F) with fly maggot protein (M) on the growth performance, antioxidant levels, immune indexes, and fecal microflora in weaned blue foxes (*Alopex lagopus*). Twenty weaned blue foxes were randomly assigned to the control (F diet; 6% of F) or experimental (M diet; F substituted by M) group (10 blue foxes per group). The duration of the trial was 28 days. The results showed that there was no significant difference in average daily gain between group M and group F during the experiment (*p* = 0.473). Moreover, the diarrhea index was similar between group M and group F during the entire experimental period (*p* = 0.112). At the end of the experiment, the levels of IL-6 and IgG in group M at 28 d were significantly higher than that in group F (*p* = 0.004, *p* = 0.025, respectively), but not IL-1β, IL-2, SIgA, IgM, and TNF-α. The levels of SOD in group M at 28 d were significantly higher than those in group F (*p* = 0.001), and no difference of MDA and T-AOC was found between group F and M (*p* = 0.073, *p* = 0.196, respectively). In both groups, the diversity of fecal microbes first increased and then decreased with the progress of the experimental period. Initially, there were differences in the composition of microbial communities between the two groups. However, this difference was attenuated at later stages of the experimental period. In conclusion, fly maggot protein can replace fish meal as a source of animal protein in feed material for blue foxes during the weaning period.

## 1. Introduction

The cost of fish meal has increased in recent years. Therefore, the need for alternative protein sources has increased, prompting research into non-conventional feed ingredients. So far, insect protein has shown great promise as an alternative protein source [1,2]. Insects require less space for breeding, have short reproduction cycles, consume a wide range of food, and exhibit high conversion rates. Further, the negative environmental impact of insect breeding is much smaller than that of livestock breeding [3]. Moreover, insect protein has a high nutritional value [4]. Therefore, insect protein has gradually become a research hotspot for protein alternatives in animal feed [5]. A variety of insects have been used to derive feed ingredients, with *Hermetia illucens* [6], *Tenebrio* *molitor* [7,8], and *Musca domestica* [9] showing the highest potential.

Fly maggot protein comes from maggots cultured in a medium composed of microorganisms, milk powder, bean products, brown sugar, and starch. Fly maggot protein feed is affordable and costs less than fish meal, which is becoming increasingly more expensive. It contains a variety of amino acids, including essential amino acids required for animal growth [10]. Similar to fish meal, fly maggot protein is a high-quality animal protein source [11]. Compared to the broiler (*Gallus gallus*) fed the basal diet, feeding diets containing 10–15% fly maggots can improve the carcass quality and growth performance of broiler chickens [12]. Song et al. found that a feeding diet with 4.7–6.4% of fly maggot powder significantly improved the growth performance, immune indexes, and antioxidant enzyme activity in broiler chickens [13]. In Wang’s study, a dietary maggot meal was also proved to be positively influential in flesh quality, and replacing 270 g∙kg^−1^ fish meal did not have an impact on the growth performance and ingredient utilization of tilapia (*Oreochromis niloticus*) [14].

The scale of blue fox (*Alopex lagopus*) breeding is increasing steadily. Blue foxes are carnivorous canines; hence, in captivity, most of the animal protein in their diet comes from fish meal. Accordingly, there is a high demand for fish meal in the blue fox breeding industry [15,16]. However, there are little data on the use of insect protein sources as a feed alternative for the blue fox diet. Studies examining the effects of insect protein on growth and plasma parameters have only been conducted on dogs [17]. Furthermore, information on the microbiome of this species is very limited, although studies indicate that insect meal can positively influence the microbiota composition and improve the gastrointestinal health in these animals [18,19].

Accordingly, this study aimed to evaluate the effect of replacing fish meal with fly maggot protein feed on growth, the blood immune indexes, and the intestinal microbiota in weaned blue foxes. The results of the present study could contribute to explain the value of fly maggot protein as a substitute for fish meal in weaned blue fox diets.

## 2. Materials and Methods

Our research was conducted in accordance with the guidelines of the Animal Welfare and Ethics Committee of Northeast Agricultural University. All the protocols were approved by the Northeast Agricultural University Animal Care and Use Committee.

### 2.1. Animal Diets, Management, and Experimental Design

#### 2.1.1. Animal Diets

Fish meal, expanded corn, rice bran meal, soybean meal, expanded soybean, corn gluten meal, Distillers Dried Grains with Solubles (DDGS), meat and bone meal, feather meal, poultry fat, limestone, sodium chloride, lysine, methionine, and premix were purchased from commercial animal feed retailers. Fly maggot protein was purchased from Guangdong xintai biotechnology Co., Ltd. (Jiangmen, China), and the content of crude protein was 54.75%. The fish meal (F) diet and fly maggot protein (M) diet were made into a powdered diet according to Table 1 by Shenyang Shuangliang Feed Co., Ltd. (Shenyang, China). Before feeding, powdered diet was mixed with an appropriate amount of water into paste.

#### 2.1.2. Animal Management and Experimental Design

The experiment was conducted in the arctic blue fox breeding base of Tuqiang Forestry Bureau in Mohe city, Daxing’anling region. Twenty weaned blue foxes (45 days of age, average body weight of 2.06 ± 0.30 kg) were assigned to a normal fish meal (F) diet group or a fly maggot protein (M) diet group, a single-factor randomized design (10 foxes per group). In the M diet group, fish meal was replaced with fly maggot protein (Table 1). Each group consisted of ten replicates, and the trial lasted for 4 weeks (1 week = 1 trial phase). In order to exclude the influence of uneven sunshine on the experiment, all experimental animals were moved to single cages on the south side. The animals were fed twice a day, once at 08:30 and once at 18:30, and had free access to water. The entire experiment was managed by fixed trained personnel.

### 2.2. Sample Collection

On days 0, 7, 14, 21, and 28 of the experimental period, body weight (BW) was measured in the morning before feeding to calculate the average daily gain (ADG), and stool samples were also collected with sterile cotton swab from rectum and stored at −80 °C. Finally, blood samples were collected through hindlimb venipuncture and centrifuged at 3500 rpm for 10 min. The serum was separated and added to 1.5 mL centrifuge tubes and stored at −20 °C until further analysis. Biochemical indexes—including immunoglobulin-G (IgG), immunoglobulin-M (IgM), tumor necrosis factor-α (TNF-α), interleukin-1β (IL-1β), interleukin-2 (IL-2), Interleukin-6 (IL-6), malondialdehyde (MDA), superoxide dismutase (SOD), total antioxidant capacity (T-AOC), and secreted immunoglobulin-A (SIgA)—were evaluated using commercial kits according to the manufacturers’ instructions (Nanjing Jiancheng ELISA kit). Meanwhile, the stool samples were sent for Illumina sequencing. Instances of diarrhea (i.e., loose stools or fecal adhesions around the anus) were independently observed by two observers at 08:30 and 18:30 daily (Table 2). The incidence of diarrhea and the diarrhea index in each group were calculated using statistical scores.

### 2.3. DNA Extraction and 16S rRNA High-Throughput Amplicon Target Sequencing

#### 2.3.1. DNA Extraction and PCR Amplification

Steps of DNA extraction: (1) Added 0.25–0.5 g of sample to a 2 mL centrifuge tube. Subsequently, added 500 µL Buffer SA, 100 µL Buffer SC, and 0.25 g grinding beads. Shook and mixed the solution with a tissue homogenizer and heated it at 70 °C for 15 min to improved lysis efficiency. Centrifuged the mixture at 12,000 rpm for 1 min, and then transferred the supernatant (about 500 µL) to a new 2 mL centrifuge tube. (2) Added 200 µL of buffer SH and mix, vortexed for 5 s, and placed at 4 °C for 10 min. (3) Centrifuged at 12,000 rpm for 3 min. Then, transferred the supernatant to a new 2 mL centrifuge tube, added 500 µL buffer GFA, inverted and mixed. (4) Added 10 µL Magnetic Bead Suspension G, shook and mixed for 5 min. (5) Placed the centrifuge tube on the magnetic stand for 30 s. After the magnetic beads were completely adsorbed, carefully removed the liquid. (6) Removed the centrifuge tube from the magnetic stand, added 700 µL of protein removal solution RD, and shook and mixed for 5 min. (7) Placed the centrifuge tube on the magnetic stand for 30 s. After the magnetic beads were completely absorbed, carefully removed the liquid. (8) Removed the centrifuge tube from the magnetic stand, added 700 µL of rinse solution PWD, and shook and mixed for 3 min. (9) Placed the centrifuge tube on the magnetic stand for 30 s. After the magnetic beads were completely adsorbed, carefully removed the liquid. (10) Repeated steps 8 and 9 once again. (11) Placed the centrifuge tube on a magnetic stand and dried at room temperature for 5–10 min. (12) Removed the centrifuge tube from the magnetic stand, added 50–100 µL of elution buffer TB, shook and mixed. Placed the tube at 56 °C, incubated for 5 min. During this period, shook and mixed the tube thrice (3–5 times each time). (13) Placed the centrifuge tube on a magnetic stand for 2 min. After the magnetic beads were completely adsorbed, carefully transferred the DNA solution to a new centrifuge tube and stored it under appropriate conditions.

PCR (polymerase chain reaction) amplification: Universal primers (338F 5′-ACTCCTACGGGAGGCAGCA-3′; 806R 5′-GGACTACHVGGGTWTCT)-3′ were used to amplify the 16S rDNA fragment V3 and V4 regions. Each specimen was amplified using specific primers marked with a barcode. To ensure the accuracy and reliability of subsequent data analysis, two conditions had to be met: ① low cycle number amplification and ② equal number of amplification cycles for each sample. Representative samples for pre-experimentation were randomly chosen to ensure that the majority of samples could be amplified using the appropriate product concentration and the minimum number of cycles. PCR amplification of the V3–V4 variable regions was performed using TransStart FastPfu Fly DNA Polymerase with a 10 µL reaction system. The reaction program was as follows: pre-denaturation at 95 °C for 5 min; 25 cycles of denaturation at 95 °C for 30 s, annealing at 50 °C for 30 s, and extension at 72 °C for 40 s; and a final extension at 72 °C for 7 min.

The PCR products were recovered with 1.8% agarose, purified with the AxyPrep DNA Gel Extraction Kit, eluted with Tris-HCl, and detected by 1.8% agarose electrophoresis. Detection and quantification were performed using QuantiFluorTM-ST.

#### 2.3.2. Illumina MiSeq

A PE 2*300 library was constructed using purified amplified fragments according to the standard operating procedures of the Illumina MiSeq platform. MiSeq library construction: (1) Connect “Y”-shaped adapters. (2) Use magnetic bead screening to remove adapter self-ligating fragments. (3) Use PCR amplification to enrich library templates. (4) Perform sodium hydroxide denaturation to generate single-stranded DNA fragments. Miseq sequencing: (1) One end of the DNA fragment is complementary to the primer base and fixed on the chip. (2) The DNA fragment is used as the template, and the base sequence fixed on the chip is used as the primer for PCR synthesis. The target is synthesized on the chip. During the procedure, DNA fragments are detected. (3) After denaturation and annealing, the other end of the DNA fragment on the chip is randomly complementary to another nearby primer, and is also fixed to form a “bridge”. (4) Perform PCR amplification to generate DNA. (5) DNA amplicons are linearized into single strands. (6) Add modified DNA polymerase and dNTPs with 4 fluorescent labels with only one base being synthesized per cycle. (7) Using laser scanning read the nucleotide species polymerized in the first round of the reaction for each template sequence. (8) Chemically cut the “fluorophore” and “termination group” to restore the viscosity of the 3′ end, and continue the polymerization for the first step. (9) Count the fluorescence signal results collected in each round to determine the sequence of the template DNA fragment.

#### 2.3.3. Processing of Sequencing Data

The sequence data were obtained after the library was built according to the relevant process of the Illumina Miseq platform and sequenced on the machine. Valid sequences were obtained by distinguishing between samples based on the barcodes and primer sequences at both ends of the sequence. Subsequently, sequence direction was corrected, that is, the optimized data were sequenced on the platform to obtain the original off-machine data. Next, before biological analysis, in order to ensure the reliability of the analysis results and ensure the quality of the sequence, further data removal was conducted. The specific requirements were as follows: ① Filtered the bases with a quality value of less than 20 in the tail of the reads, and set a window of 50 bp. If the average quality value in the window was lower than 20, we truncated the back-end bases from the window, filter reads below 50 bp after quality control, and removed reads containing N bases. ② According to the overlapping relationship between reads, we spliced reads into one sequence (minimum overlap length is 10 bp). ③ The maximum mismatch ratio allowed in the overlap region of the spliced sequence is 0.2, and the non-conforming sequences are screened. ④ We distinguished between samples based on the barcodes and primers at the beginning and end of the sequence samples, and adjusted the sequence orientation; the barcode does not allow mismatches. Through clustering, the sequences were divided into many groups based on sequence similarity; these groups were called operational taxonomic units (OTUs).

### 2.4. Statistical Analysis

The original data were preliminarily processed by Excel. The average daily weight gain, the diarrhea rate, and the diarrhea index were calculated according to the following formula:

Average daily gain (ADG) (g/day) = (final weight-initial weight) (g)/feed days (day)

The diarrhea rate = number of blue foxes with diarrhea/total number of test blue foxes × 100%

Diarrhea index=∑diarrhea scoring/total number of test blue foxes

ADG, diarrhea rate, and diarrhea index were tested for normal distribution. The T-test program in SPSS 23.0 statistical software was used for analysis of ADG, diarrhea rate, diarrhea index, immune indexes, and serum antioxidant indexes, and the results are expressed as mean ± standard deviation. Significance was set at *p* < 0.05.

All sequences could be divided into OTUs according to different similarity levels. Usearch (version 10.0) was used to cluster the sequences at a similarity level of 97% (default). By default, 0.005% of the number of sequenced sequences was used as the threshold to filter OTUs. The RDP classifier (http://rdp.cme.msu.edu/) (accessing on 20 November 2021) was used to perform species annotation for each sequence based on comparisons with the Silva (Release128 http://www.arb-silva.de) (accessing on 20 November 2021) database. The alignment threshold was set to 70%. QIIME2 (https://qiime2.org/) (accessing on 20 November 2021) was used for alpha index analysis, including rarefaction curve, Shannon curves, ACE richness estimator, Chao1 richness estimator, Simpson diversity index, Shannon diversity index, and Rank abundance curve. Principal component analysis (PCA) was used to sort through a series of eigenvalues and eigenvectors and was performed based on R language platform. Lefse was performed based on online platform (http://huttenhower.sph.harvard.edu/lefse/). (accessing on 20 November 2021). The threshold for statistical significance was *p* < 0.05.

## 3. Results

### 3.1. Effects of Fly Maggot Protein Replacing Fish Meal on Growth Performance of Weaned Blue Foxes

As shown in Table 3, the average daily weight gain in group F was significantly higher than that of in group M at 0–7 d (*p* = 0.001). However, there was no significant difference in the average daily weight gain between the groups at 8–14 d and 22–28 d (*p* = 0.276; *p* = 0.894, respectively). Between 15 and 21 d, the average daily weight gain in group M was significantly higher than that in group F (*p* = 0.002). During the whole experimental period, there was no significant difference between the body weight and the average daily gain between the two groups (*p* > 0.05).

During the whole experimental period, the diarrhea rate in group M was significantly higher than that in group F (*p* = 0.047), but the diarrhea index was not significantly different between the two groups (*p* = 0.109). In group M, the diarrhea rate decreased gradually as the experiment progressed (Table 3).

### 3.2. Effects on Immune Indexes

The effects of replacing fish meal with fly maggot protein feed on immune parameters in weaned blue foxes are illustrated in Table 4. IL-1β levels in group M were significantly higher than those in group F at 21 d (*p* = 0.013), but there was no significant difference between these groups at 28 d (*p* = 0.074). At 0 and 7 d, the levels of IL-2 in group M were significantly higher than those in group F (*p* = 0.002 and *p* = 0.005, respectively). At 21 d, the levels of IL-2 in group M were significantly higher than those in group F (*p* = 0.022). At 21 and 28 d, the levels of IL-6 in group M were significantly higher than those in group F (*p* = 0.004 and *p* = 0.049, respectively). On day 7, the IgM level in group M was significantly higher than that in group F (*p* < 0.001), but there was no significant difference between the two groups at the other timepoints (*p* > 0.05). The levels of TNF-α, IgG, and SIgA were similar between the two groups throughout the experiment (*p* > 0.05).

### 3.3. Effects on Serum Antioxidant Indexes

The effect of replacing fish meal with fly maggot protein feed on serum antioxidant indexes in weaned blue foxes is illustrated in Table 5. No significant differences in MDA levels were observed between the two groups throughout the whole experimental period (*p* > 0.05). However, the SOD levels in group F were significantly higher than those of group M (*p* < 0.01), but at 14 d, this value was significantly higher than in group F (*p* < 0.05). The concentration of T-AOC in group F was significantly higher than that in group M at 0, 7, 14, and 21 d (*p* < 0.05, *p* < 0.01, *p* < 0.05, and *p* < 0.01, respectively). However, there was no significant difference in this index between the two groups at 28 d (*p* > 0.05).

### 3.4. Effects of Fly Maggot Protein Substitute for Fish Meal on Fecal Flora of Blue Foxes in Weaning Period

**Figure 1:**
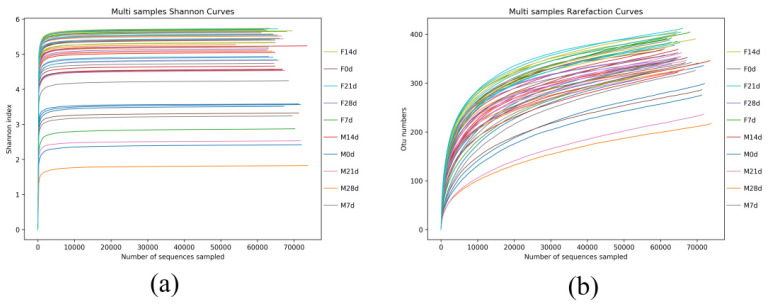
All samples Shannon index curve and Rarefaction curve. (**a**): Shannon curves of the multi samples; (**b**): Rarefaction curve of the multi samples.

Based on a 97% sequence similarity, the identified sequences were clustered into OTUs [20]. Figure 1 shows the number of OTUs in each sample obtained after clustering. The number above the column represents the number of OTUs in the corresponding sample.

To determine whether the sample sequencing depth was sufficient for subsequent analysis, rarefaction and Shannon curves were plotted (Figure 1). The rarefaction curve did not reach a plateau, indicating that new species could be discovered if the sequencing volume was increased. However, the Shannon curve showed that all samples had entered the plateau phase. Hence, at the current sequencing depth, this study captured the vast majority of species in the samples. Therefore, the data quality was sufficient for subsequent analysis.

#### 3.4.1. Sample Diversity Analysis

The Shannon index, Simpson index, Chao1, and ACE were used to evaluate the Alpha diversity of fecal samples from weaned foxes reared on different protein sources (Table 6). Protein replacement with fly maggot feed led to significantly lower Shannon index values at 0 d (*p* < 0.05) and 14 d (*p* < 0.01). The Chao1, Simpson, and ACE indexes also tended to be lower.

#### 3.4.2. Venn Diagram Analysis Based on OTUs

**Figure 2:**
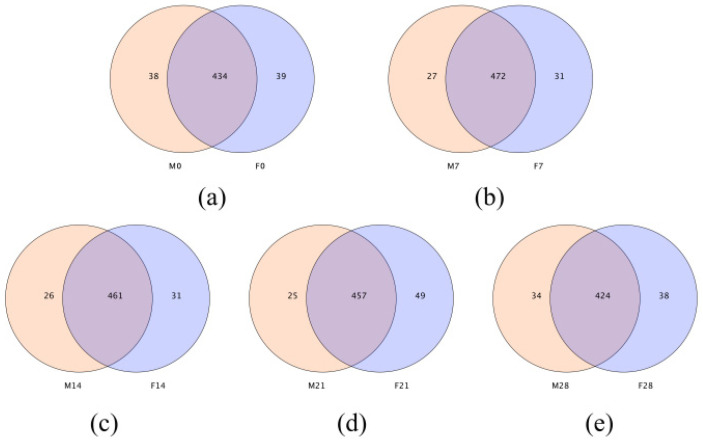
OTU-based Venn diagram analysis: the effect of different protein sources for fish meal substitution on the fecal flora of blue foxes. (**a**): Venn diagrams of the fecal flora between M0-vs-F0; (**b**): Venn diagrams of the fecal flora between M7-vs-F7; (**c**): Venn diagrams of the fecal flora between M14-vs-F14; (**d**): Venn diagrams of the fecal flora between M21-vs-F21; (**e**): Venn diagrams of the fecal flora between M28-vs-F28.

Venn diagrams can more intuitively reflect the similarity and overlap of sample OTU composition. Overall, 434, 472, 461, 457, and 424 OTUs were identified in the two groups of foxes at 0 d, 7 d, 14 d, 21 d, and 28 d, respectively. The number of OTUs shared by the two groups first increased with the experimental period and then decreased. The number of unique OTUs in groups F and M was 39 and 38, respectively, at 0 d. This number was 31 and 27, respectively, at 7 d; 31 and 26, respectively, at d 14; 49 and 25, respectively, at d 21; and 38 and 34, respectively, at d 28. In both groups, the diversity of fecal microbes first increased and then decreased with the progress of the experimental period, consistent with the results of alpha diversity analysis (Figure 2).

#### 3.4.3. Multi-Sample Comparative Analysis

**Figure 3:**
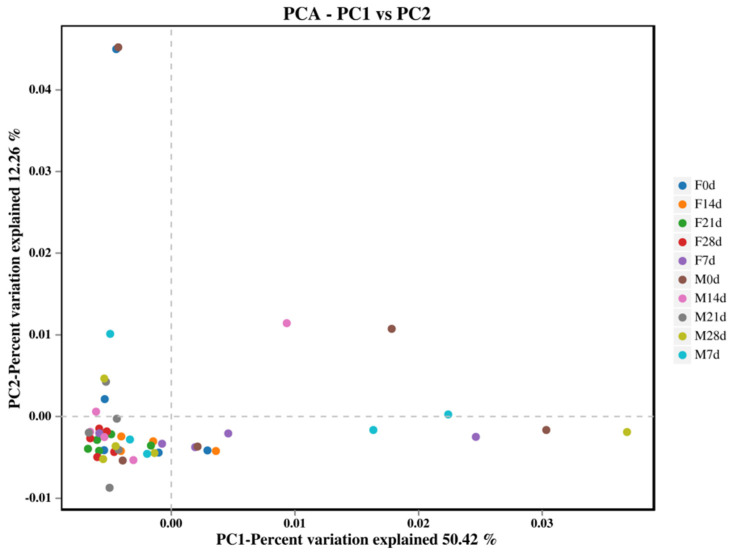
Similarity of fecal microbiota composition between weaned foxes reared on fish meal or a fly maggot protein replacement at different stages during the experimental period.

Based on abundance information and the species annotation of all sample OTUs, principal component analysis (PCA) was performed to analyze the fecal samples of the two groups of weaned foxes at five time points (Figure 3). The contributions of the first and second principal components to the sample differences were 50.42% and 12.26%, respectively. Although some samples were scattered on the score map, the distribution of most samples was more concentrated. This indicates that there were no obvious structural differences between the microbes in the fecal samples collected from the two groups at different time points.

#### 3.4.4. Structural Analysis of Fecal Flora

**Figure 4:**
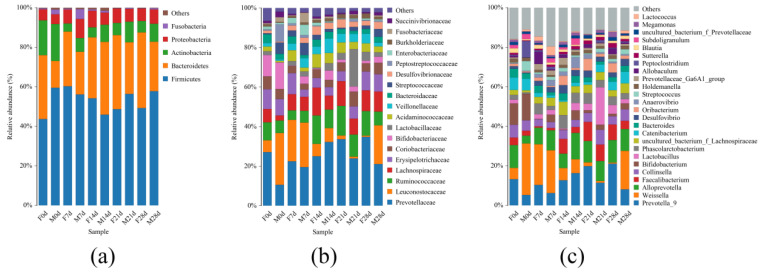
Relative abundance of microorganisms in fecal samples from blue foxes reared on different protein sources at the phylum (**a**), family (**b**), and genus (**c**) levels.

We studied the microbial community structure in the collected fecal samples. Five dominant bacterial phyla (mean relative abundance > 1%) were identified (Figure 4a): *Firmicutes*, *Bacteroidetes*, *Actinobacteria*, *Proteobacteria*, and *Fusobacteria*. The abundance of *Bacteroidetes* in group F was significantly higher than that in group M on day 0 (*p* < 0.01). With age, the abundance of *Bacteroidetes* in group M increased, and eventually, there was no significant difference in this value between the two groups. However, the overall abundance of *Bacteroidetes* in group F was higher than that in group M (*p* > 0.05). After day 0, the relative abundance of *Actinobacteria* in the two groups remained low, but the relative abundance in group M was still higher than that in group F (*p* > 0.05).

In total, 18 families with a mean relative abundance > 1% were identified. These 18 families accounted for 97.06% of all microbes identified (Figure 4b). *Prevotellaceae* (25.02%), *Leuconostocaceae* (11.19%), *Ruminococcaceae* (9.55%), *Lachnospiraceae* (8.70%), and *Erysipelotrichaceae* (7.10%) were the most abundant families across all samples, accounting for 61.56% of all sequences. In group M, the abundance of *Prevotellaceae* at 0 d was significantly lower than that in group F (*p* < 0.01). There was no significant difference in the relative abundance of *Prevotellaceae* between group F and group M at the further time points. The relative abundance of *Lachnospiraceae* in group F at 14 d was significantly higher than that in group M (*p* < 0.01). Although the abundance of these bacteria tended to be higher in group F at all other time points as well, the difference was not significant.

In total, 25 genera with a mean relative abundance > 1% were identified. These 25 genera accounted for 86.22% of all sequences (Figure 4c). *Prevotella_9* (12.49%), *Weissella* (10.99%), and *Alloprevotella* (9.81%) were the most abundant genera across all samples, accounting for 33.29% of all sequences. The relative abundance of *Faecalibacterium* in group F was higher than that in group M; this difference became significant at d 14 (*p* < 0.05). The relative abundance of *uncultured_bacterium_f_Lachnospiraceae* in group F was significantly higher than that in group M (*p* < 0.01), but there was no significant difference at other time points. The relative abundance of *Oribacterium* in group F was significantly higher than that in group M at 21 d and 28 d (*p* < 0.05). However, the other genera showed no significant difference between the two groups during the entire experimental period (*p* > 0.05).

#### 3.4.5. LEfSe Species Difference Analysis

**Figure 5:**
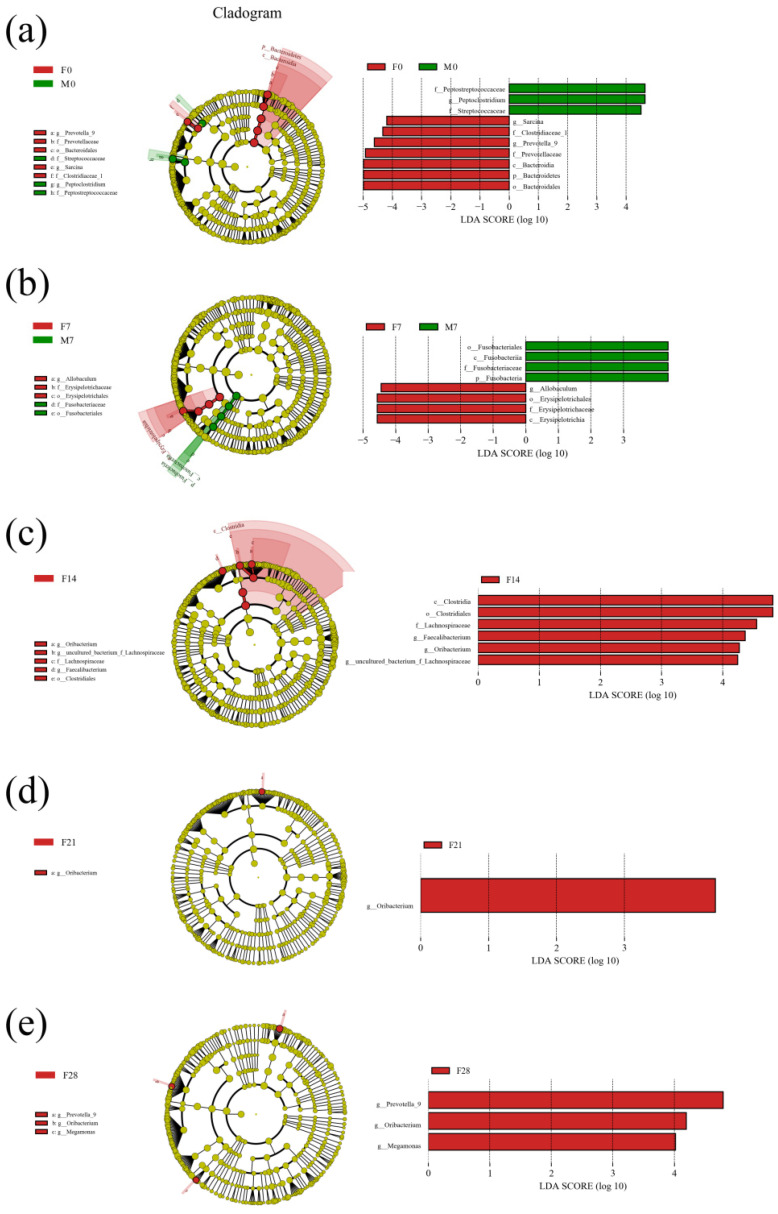
LDA analysis of fecal flora of two groups of blue foxes on 0 d (**a**), 7 d (**b**), 14 d (**c**), 21 d (**d**), and 28 d (**e**) of the experimental period.

Comparisons between groups F and M were made at different time intervals during the intervention period. To identify the differentially abundant taxa, the LDA of the effect size was performed based on a threshold score of 4.0. Bacterial clades were identified at five different time points (Figure 5). At 0 d, the relative abundance of 10 bacterial clades was significantly different between the two groups; 7 of these were enriched in group F, whereas 3 were enriched in group M. At 7 d, the relative abundance of eight bacterial clades was significantly different between the two groups; four of these were enriched in group F, and four were enriched in group M. At other time points, there were no bacterial clades that significantly distinguished group M from group F. The results indicated that initially, there were differences in the composition of microbial communities between the two groups. However, this difference was attenuated at later stages of the experimental period.

## 4. Discussion

Fly maggots contain 59–65% crude protein [21] and are a high-quality source of animal protein, comparable to fish meal [22]. Fly maggot protein is known to improve growth in weaned piglets (*Sus scrofa*), reducing the cost of growth and making pig breeding more economic [23]. A study showed that when Chinese soft-shelled turtles (*Pelodiscus sinensis*) were fed an appropriate amount of fly maggot protein, their feed coefficient effectively reduced, while their growth, immune indexes, and survival rates improved [24,25,26]. Replacing fish meal with fly maggot meal not only improves growth and survival rates in tilapia but also increases their immunity and crude protein content [5,27]. These studies together indicate that the use of fly maggot protein as a feed supplement or animal protein alternative has no negative effect on growth and improves production efficiency.

In this study, the replacement of fish meal with fly maggot protein had a significant effect on the average daily weight gain of weaned foxes at various time points. However, when the entire experimental period was considered, no significant difference in this value was observed. The body weight of group M was higher than that of group F at 7 d and 14 d but lower at 21 d and 28 d, although the differences were not significant. Therefore, when weaned blue foxes grow to a certain stage, replacing fish meal with fly maggot protein may provide a better feeding effect and improve their growth. The experimental results suggest that fly maggot protein can replace fish meal for blue foxes at the weaning stage and reduce the cost of breeding.

In addition, although the incidence of diarrhea in group M was significantly higher than that in group F, there was no significant difference in the diarrhea index. The reasons for the high incidence of diarrhea may be as follows: (1) Monogastric animals may find it difficult to digest the chitin in insect proteins. Chitin has an anti-nutritional effect and can reduce protein utilization to a certain extent [28,29]. (2) The ratio of fish meal replacement is not optimal, and excessive addition of insect protein may have negative effects on animals [30]. (3) The experiments were conducted on weaned foxes, who may have digestive organs that are not completely developed. Although the change in feed ingredients does not affect palatability, it may affect digestion in young blue foxes. (4) The stress of weaning and separation from companions may lead to disordered bodily functions in young blue foxes. However, our findings indicated that fly maggot protein can reduce the incidence of diarrhea in blue foxes at the middle and late stages. Therefore, further research is needed to determine the proportion of fish meal replacement with insect protein and the optimal feeding time to maximize the benefits of protein replacement.

IgG, IgA, and IgM are the main effector molecules mediating humoral immunity and reflect the immune status of the body [31]. IgG has antiviral and antitoxin activities in vivo and is the most important immunoglobulin in humoral immunity [32]. IgM is involved in intestinal immunity. The IgM levels in group M were always higher than those in group F, even at the early stages of the experiment. This may be because the fly maggot protein improved the immune status of young blue foxes during the pre-feeding period. Therefore, the IgM content on d 0 was already higher than that in group F, and this difference was maintained during the subsequent test period. IgA represents an important line of defense against the adhesion and colonization of pathogens in the intestinal mucosa, and it is also the most common and most secreted immunoglobulin in the body [33]. In this study, there was no significant difference in IgA levels between group M and group F. This indicated that the replacement of fish meal with fly maggot protein did not create any immune stress in blue foxes during the weaning period.

Weaned blue foxes are prone to immune stress for the following reasons: discontinuation of breast milk, immature immune system, presence of protein antigens in the feed, and environmental changes. During conditions of immune stress, the levels of IL-1β, IL-6, and TNF-α increase. Instead of being used to maintain growth, the nutrients in the body are used to mount an immune response, inhibiting growth and metabolism. IL-1 is an important cytokine secreted by macrophages. Its excessive secretion can stimulate a variety of immune cells and inflammatory cells, thereby leading to the productions of cytokines, such as IL-6 and TNF-α, that induce inflammatory responses and cell damage. IL-2, an important factor in the series of lymphokines released during an immune response, is considered a marker of T cell activation. Our study found that the level of IL-2 was significantly higher in the fly maggot protein group than in the control group, while no significant difference in TNF-α was observed.

Therefore, the results of this study indicate that feed protein may cause stress responses in young foxes. The effects of fly protein feed were clearly more positive than those of fish meal. This is consistent with findings from studies on weaned piglets, loaches, and poultry [23,29,34]. Fly maggot protein contains bioactive substances, such as fly maggot chitosan, fly maggot peptide, and lectin, which may improve immune function in young blue foxes.

Redox reactions are normal physiological and biochemical reactions and can lead to the generation of free radicals. MDA is an important product of lipid peroxidation and indicates the rate and intensity of lipid peroxidation. Thus, it can be used to indirectly estimate oxidative damage [35]. T-AOC reflects the overall activity of enzymatic and non-enzymatic antioxidant systems [36]. It is usually used to directly examine the activity of antioxidant enzymes in the body. SOD and CAT are the most important antioxidant enzymes and serve as a defense system against O^2−^ radicals. SOD can prevent excessive O^2−^ invasion and its activity is an indirect indicator of oxidative damage.

Antioxidant systems of organisms are affected differently by feeding insect proteins. Studies have shown that 4.7–6.4% of fly maggot protein in feed can positively influence immunity and antioxidant capacity in broiler chickens [11]. However, other studies have found different results. The CAT levels in Nile tilapia were not significantly affected after feeding with fly maggot protein [37]. Hence, the response to different protein sources can vary from species to species, and the defense strategies may also vary. In this experiment, the levels of SOD and T-AOC in group M had no significant difference in the later stage of the experiment. MDA levels did not show significant between-group differences throughout the experimental period. These findings suggest that feeding fly maggot protein to weaned blue foxes has no negative effect on their antioxidant capacity.

Microbes play an important role in the development of the intestinal mucosal system and tissues [38]. Microbial colonization is a complex process. Each host develops a unique bacterial community structure through long-term evolution, which is maintained in a state of equilibrium via interaction with environmental factors. Moreover, there are significant differences in the microbiota between species. Various factors, such as maternal microflora composition, age, dietary changes, and antibiotics, all influence gut microflora composition [39]. In this experiment, the Shannon index of group M was significantly lower than that of group F on day 0. However, no significant difference in ACE, Chao1, Simpson, and Shannon index values of the fecal microflora was observed between the two groups at 28 d. Hence, after feeding with fly maggot protein, the richness of the microbial community in weaned foxes could be increased, although the diversity remains unaffected.

The genus *Prevotella* contains highly active bacteria that decompose hemicellulose [40]. As part of the intestinal microflora, these bacteria play an important role in the degradation of hemicellulose and promote the degradation of non-structural carbohydrates [41,42], improving digestion and absorption. Hence, they have a positive impact on health. *Prevotella* spp. play a key role in the utilization of starch, xylan, and pectin during digestion in cattle and sheep [43,44,45]. In addition, *Prevotella* can degrade plant cell walls [42]. The abundance of *Prevotella* in the rumen is associated with fiber digestibility [46], and these bacteria also secrete proteases [47]. Although *Prevotella* is abundant in blue fox feces, the exact role of these bacteria in blue foxes remains to be determined. Blue foxes show a high utilization rate of protein- and fat-rich animal feed [48]. However, whether and how their intestinal bacteria participate in the metabolism of nutrients, such as protein and fat, is still unclear.

Lactic acid bacteria produce a large amount of lactic acid, which can reduce the pH of the intestinal tract. These bacteria can also produce bacteriostatic metabolites and thereby, inhibit the reproduction and invasion of pathogenic bacteria. Therefore, lactic acid bacteria have a positive impact on immunity and intestinal health [49]. *Weissella* spp. are present in most fermentation products [50,51]. In the early stage of fermentation, *Weisseria* can even dominate in numbers. At this stage, many species of *Weisseria* have been found to have the advantages of probiotics, with various probiotic properties, including: the production of bacteriocins, which have been found to inhibit pathogenic bacteria [52]; prevent it from adhering to cells [53]; produce exopolysaccharides, such as glucan, etc. [54,55,56]; generate digestive enzymes [57]; and have antioxidant capacity [52,58,59,60].

Consistent with Chen’s findings, this study showed that the fecal flora in weaned foxes mainly consists of *Firmicutes*, *Bacteroidetes*, *Actinobacteria*, *Proteobacteria*, and *Fusobacteria*. Together, these bacteria account for 97% of the microbiota [61]. At the family level, *Prevotellaceae* was the dominant bacterial group. No significant difference in the relative abundance of *Prevotellaceae* was observed between groups M and F overall, although this value was higher in group F on day 0. At the genus level, *Weissella* and *Prevotella_9* made up the dominant flora. The presence of fly maggot protein in feed increased the relative abundance of *Weissella* in weaned blue foxes, although the difference was not significant. In contrast, blue foxes consuming fish meal had a significantly higher relative abundance of *Prevotella_9*. The effect of fly maggot protein on the relative abundance of other fecal microbes was not significant. However, the abundance of each group showed different trends of change over time. This suggests that when fly maggot protein was used to replace fish meal, there was no adverse effect on the structure of the fecal flora. Hence, fly maggot protein could replace fish meal in the diet of blue foxes during the weaning period.

## 5. Conclusions

The replacement of fish meal feed with fly maggot protein can affect some indicators (such as antioxidant levels and immunity) in blue foxes at the weaning stage. However, over time, growth, immunity, and antioxidant levels tend to stabilize and become similar to those in blue foxes receiving fish meal. Moreover, fly maggot protein does not significantly alter the fecal microbial community. Moreover, the effects of fly maggot protein replacement improve with time. Therefore, fly maggot protein can be used as an alternative protein source for blue foxes, instead of expensive fish meal, during the weaning period.

There are some limitations to this study. The effect of fly maggot protein on blue foxes was examined during the weaning period. However, the optimal age or period for fly maggot protein replacement could not be determined. Future studies should explore this aspect to maximize the production efficiency of blue foxes and reduce costs.

## Figures and Tables

**Table 1:** Feed formula and nutritional composition.

Ingredient (DM%)	Groups	
	Fish Meal	Fly Maggot Protein
Expanded corn	36.98	36.97
Rice bran meal	2.70	0.50
Soybean meal	19.99	19.98
Expanded soybean	3.00	3.00
Corn gluten meal	5.00	6.50
Distillers Dried Grains with Solubles	10.00	9.99
Fish meal	6.00	-
Fly maggot complex protein	-	6.00
Meat and bone meal	6.00	6.00
Feather meal	5.00	5.00
Poultry fat	4.00	4.00
Limestone	0.00	0.40
Sodium chloride	0.05	0.16
Lysine	0.00	0.16
Methionine	0.30	0.35
Premix ^1^	1.00	1.00
Total	100.00	100.00
Nutrient levels ^2^ (DM%)		
Crude protein	29.90	29.92
Crude fat	8.47	8.36
Ash	6.15	5.99
Total phosphorus	0.89	0.72
Available phosphorous	0.64	0.51
Ca	1.07	0.99
Lysine	1.42	1.40
Methionine	0.79	0.79
Salt	0.43	0.45

^1^ Premix per kg of diet: iron, 80 mg; zinc, 60 mg; manganese, 15 mg; copper, 10 mg; iodine, 0.50 mg; selenium, 0.20 mg; cobalt, 0.30 mg; vitamin A, 10,000 IU; vitamin B_1_, 20 mg; vitamin B_2_, 10 mg; Vitamin B_6_, 10 mg; Vitamin B_12_, 0.10 mg; VC, 120 mg; Vitamin E, 60 mg; Vitamin K_3_, 1.60 mg. ^2^ Metabolizable energy is the calculated value, the rest is the measured value.

**Table 2:** Diarrhea scoring criteria.

Score	Diarrhea	Fecal State
0	Normal	Stools come in streaks or pellets
1	Mild	Forming, soft stools
2	Moderate	No separation of feces and water, thick and loose stools
3	Severe	Feces are liquid, formless, the feces are separated from water

**Table 3:** Effects of different protein sources replacing fish meal on the growth performance of weaned blue foxes.

Items	Groups		*t*-Value	*p*-Value
	Fish Meal	Fly Maggot Protein		
Day 0 BW/kg	1.96 ± 0.24	2.04 ± 0.28	−0.730	0.475
Day 7 BW/kg	2.47 ± 0.20	2.33 ± 0.22	1.488	0.154
Day 14 BW/kg	2.92 ± 0.17	2.74 ± 0.29	1.788	0.091
Day 21 BW/kg	3.42 ± 0.22	3.45 ± 0.30	−0.254	0.802
Day 28 BW/kg	4.05 ± 0.20	4.08 ± 0.34	−0.199	0.845
Days 1 to 7				
Average daily gain/g	74.29 ± 11.27 ^A^	42.14 ± 22.20 ^B^	4.082	0.001
Diarrhea%	10.71 ± 7.72 ^B^	20.00 ± 10.54 ^A^	−2.248	0.037
Diarrhea index	0.63 ± 0.18	0.81 ± 0.21	−2.051	0.055
Days 8 to 14				
Average daily gain/g	64.29 ± 17.82	57.14 ± 18.75	0.873	0.394
Diarrhea%	14.29 ± 13.47	20.71 ± 10.88	−1.174	0.256
Diarrhea index	0.64 ± 0.34	0.74 ± 0.12	−0.940	0.367
Days 15 to 21				
Average daily gain/g	70.71 ± 18.58 ^B^	102.14 ± 19.65 ^A^	−3.675	0.002
Diarrhea%	15.00 ± 10.35	15.71 ± 7.38	−0.178	0.861
Diarrhea index	0.66 ± 0.22	0.62 ± 0.17	0.404	0.691
Days 22 to 28				
Average daily gain/g	90.00 ± 13.55	89.29 ± 9.67	0.136	0.894
Diarrhea%	11.43 ± 9.04	13.57 ± 5.27	−0.648	0.525
Diarrhea index	0.65 ± 0.27	0.83 ± 0.37	−1.238	0.232
Days 0 to 28				
Average daily gain/g	74.82 ± 4.94	72.68 ± 7.81	0.733	0.473
Diarrhea%	12.86 ± 5.24 ^b^	17.50 ± 4.44 ^a^	−2.137	0.047
Diarrhea index	0.64 ± 0.17	0.75 ± 0.11	−1.687	0.112

^a,b^ Values within a row with different superscripts differ significantly at *p* < 0.05. ^A,B^ Values within a row with different superscripts differ significantly at *p* < 0.01.

**Table 4:** Effects of different protein sources in place of fish meal on immune indexes of weaned blue foxes.

Items	Groups		*t*-Value	*p*-Value
	Fish Meal	Fly Maggot Protein		
IL-1β (ng/L)				
0 d	173.06 ± 21.31	183.55 ± 6.67	−0.183	0.462
7 d	233.96 ± 13.09	223.88 ± 17.84	0.789	0.474
14 d	204.12 ± 10.91	210.97 ± 13.09	−0.697	0.524
21 d	182.74 ± 12.59 ^b^	227.10 ± 12.94 ^a^	−4.256	0.013
28 d	186.37 ± 17.71	211.78 ± 4.58	−2.405	0.074
IL-2 (pg/mL)				
0 d	563.02 ± 38.41 ^B^	800.10 ± 41.94 ^A^	−7.220	0.002
7 d	681.56 ± 53.80 ^B^	877.93 ± 28.06 ^A^	−5.605	0.005
14 d	719.88 ± 40.48	733.05 ± 16.20	−0.523	0.628
21 d	606.13 ± 30.97 ^b^	736.64 ± 54.04 ^a^	−3.629	0.022
28 d	698.32 ± 12.95	739.03 ± 45.63	−1.487	0.211
IL-6 (ng/L)				
0 d	51.80 ± 18.51	76.42 ± 37.43	−1.021	0.365
7 d	58.00 ± 20.60	63.26 ± 7.88	−0.413	0.701
14 d	63.55 ± 25.12	63.80 ± 11.32	−0.016	0.988
21 d	45.19 ± 24.46 ^b^	93.12 ± 16.85 ^a^	−2.795	0.049
28 d	40.26 ± 7.60 ^B^	93.15 ± 13.09 ^A^	−6.054	0.004
SIgA (µg/mL)				
0 d	29.44 ± 1.08	29.94 ± 1.60	−0.454	0.673
7 d	28.56 ± 1.85	30.64 ± 0.58	−1.862	0.136
14 d	27.17 ± 0.49	29.67 ± 1.64	−2.523	0.065
21 d	31.15 ± 1.06	29.95 ± 1.00	2.052	0.227
28 d	32.90 ± 1.60	30.05 ± 1.40	6.389	0.233
IgG (µg/mL)				
0 d	713.46 ± 32.37 ^b^	796.70 ± 36.09 ^a^	−2.974	0.041
7 d	715.84 ± 21.70	730.11 ± 29.20	−0.679	0.534
14 d	720.59 ± 13.51 ^B^	739.57 ± 20.90 ^A^	−4.800	0.009
21 d	677.78 ± 44.32 ^B^	721.13 ± 44.32 ^A^	−5.619	0.005
28 d	665.89 ± 46.38 ^b^	739.35 ± 21.41 ^a^	−3.508	0.025
IgM (µg/mL)				
0 d	29.35 ± 2.37	33.50 ± 0.31	−3.010	0.091
7 d	32.90 ± 1.03 ^B^	38.59 ± 0.74 ^A^	−7.787	0.001
14 d	30.06 ± 2.42	33.50 ± 0.99	−2.277	0.085
21 d	33.73 ± 2.52	35.27 ± 1.58	−0.897	0.421
28 d	33.91 ± 2.08	33.02 ± 1.04	0.661	0.545
TNF-α (ng/L)				
0 d	174.74 ± 4.19	155.13 ± 33.23	1.014	0.415
7 d	167.83 ± 31.07	145.64 ± 84.19	0.428	0.690
14 d	175.74 ± 23.98	123.28 ± 14.01	−1.406	0.233
21 d	181.11 ± 34.10	151.22 ± 7.88	1.479	0.213
28 d	192.54 ± 24.15	201.22 ± 17.79	−0.502	0.642

^a,b^ Values within a row with different superscripts differ significantly at *p* < 0.05. ^A,B^ Values within a row with different superscripts differ significantly at *p* < 0.01.

**Table 5:** Effects of different protein sources in place of fish meal on serum antioxidant indexes of weaned blue foxes.

Items	Groups		*t*-Value	*p*-Value
	Fish Meal	Fly Maggot Protein		
MDA (nmol/mL)				
0 d	103.14 ± 1.86	100.40 ± 2.80	1.413	0.230
7 d	103.05 ± 0.59	102.20 ± 2.80	0.515	0.654
14 d	107.86 ± 2.09	104.65 ± 2.16	1.849	0.138
21 d	106.64 ± 1.71	104.75 ± 2.80	0.999	0.374
28 d	111.55 ± 3.61	104.56 ± 0.75	3.283	0.073
SOD (U/mL)				
0 d	932.31 ± 17.40 ^A^	668.37 ± 14.37 ^B^	20.257	<0.001
7 d	876.39 ± 4.73 ^A^	724.30 ± 2.05 ^B^	51.160	<0.001
14 d	879.11 ± 23.54 ^A^	775.45 ± 23.06 ^B^	5.449	0.006
21 d	927.54 ± 21.88 ^A^	737.25 ± 27.25 ^B^	9.432	0.001
28 d	886.62 ± 16.41 ^A^	741.38 ± 27.78 ^B^	10.105	0.001
T-AOC (U/mL)				
0 d	171.02 ± 5.27 ^a^	154.58 ± 5.54 ^b^	3.721	0.020
7 d	169.24 ± 3.53 ^A^	150.12 ± 4.78 ^B^	5.571	0.005
14 d	166.44 ± 3.91 ^a^	152.04 ± 4.05 ^b^	4.432	0.011
21 d	165.42 ± 3.34 ^A^	152.99 ± 2.13 ^B^	9.808	0.001
28 d	165.29 ± 2.49	160.57 ± 4.65	1.548	0.196

^a,b^ Values within a row with different superscripts differ significantly at *p* < 0.05. ^A,B^ Values within a row with different superscripts differ significantly at *p* < 0.01.

**Table 6:** Effects of different protein sources on the Alpha diversity of feces of weaned blue foxes.

Items	ACE	Chao1	Simpson	Shannon
0 d				
F1	386.14 ± 23.59	397.32 ± 35.01	0.90 ± 0.08	4.82 ± 0.92 ^a^
M1	374.10 ± 20.09	376.29 ± 18.38	0.76 ± 0.14	3.53 ± 0.76 ^b^
7 d				
F7	430.59 ± 23.38	427.58 ± 21.40	0.88 ± 0.13	5.04 ± 1.22
M7	412.34 ± 13.68	413.90 ± 15.85	0.88 ± 0.11	4.67 ± 0.98
14 d				
F14	436.65 ± 10.72	437.48 ± 9.97	0.95 ± 0.01	5.53 ± 0.19 ^A^
M14	427.35 ± 36.86	419.26 ± 21.12	0.92 ± 0.02	4.95 ± 0.31 ^B^
21 d				
F21	436.19 ± 11.90	436.76 ± 10.33	0.94 ± 0.02	5.31 ± 0.39
M21	391.90 ± 46.89	387.47 ± 59.51	0.87 ± 0.12	4.49 ± 1.14
28 d				
F28	392.65 ± 11.82	406.49 ± 23.06	0.93 ± 0.02	5.29 ± 0.29
M28	359.04 ± 45.55	363.32 ± 48.99	0.83 ± 0.24	4.58 ± 1.55

^a,b^ Values within a row with different superscripts differ significantly at *p* < 0.05. ^A,B^ Values within a row with different superscripts differ significantly at *p* < 0.01.

## Data Availability

The data presented in this study are available on request. These data are not publicly available to preserve the data privacy of the commercial farm.
